# Micro- and Nanoplastics in the Human Exposome: Environmental Pathways, Kidney Toxicity, and Implications for Public Health Risk Assessment

**DOI:** 10.3390/ijms27177903

**Published:** 2026-09-04

**Authors:** Mariana Abrantes do Amaral, Orestes Foresto-Neto, Matheus Fernandes de Oliveira, Régia Caroline Peixoto Lira, Rodrigo Bueno de Oliveira, Niels Olsen Saraiva Câmara, Pablo Shimaoka Chagas

**Affiliations:** 1 Department of Immunology, Institute of Biomedical Sciences IV, University of São Paulo, São Paulo 05508-270, SP, Brazil; mariana.amaral@unifesp.br (M.A.d.A.); matheusoliveiraicb@usp.br (M.F.d.O.); niels@icb.usp.br (N.O.S.C.); 2 Nephrology Division, Department of Medicine, Federal University of São Paulo, São Paulo 05508-270, SP, Brazil; fores-to.neto@unifesp.br; 3 Department of Pathology, Genetics and Evolution, Kidney Research Center, Federal University of Triângulo Mineiro, Campus I, Uberaba 38025-350, MG, Brazil; regia.fusco@uftm.edu.br; 4 Laboratory for Evaluation of Mineral and Bone Disorders in Nephrology (LEMON), School of Medical Sciences, State University of Campinas (Unicamp), Campinas 13083-852, SP, Brazil; rbo@unicamp.br

**Keywords:** microplastics, nanoplastics, nephrotoxicity, kidney disease, environmental pollution, exposome, environmental health

## Abstract

Plastic pollution has emerged as a global environmental and public health crisis, with micro- and nanoplastics (MNPs) representing a pervasive component of the human exposome. Generated through the continuous fragmentation of larger plastic materials, MNPs exhibit physicochemical properties that influence their environmental fate, biological interactions, and toxic potential. Human exposure occurs primarily through ingestion and inhalation, facilitating particle translocation across biological barriers and systemic distribution. Experimental studies have shown that MNP exposure can disrupt mitochondrial and lysosomal function, induce oxidative stress and DNA damage, and modulate inflammatory, apoptotic, and fibrotic pathways. Although evidence supports their multiorgan toxicity, the kidney has emerged as a critical target organ due to its role in filtration and excretion. Experimental studies, often conducted at concentrations exceeding estimated environmentally relevant exposure levels, have associated renal MNP exposure with oxidative injury, apoptosis, and fibrotic remodeling, while emerging mechanistic findings suggest potential links to pro-oncogenic cellular alterations; however, these effects have not been established in humans, and their clinical relevance remains uncertain. Furthermore, the detection of plastic particles in human blood, tissues, and urine underscores the translational relevance of MNP exposure while highlighting important gaps in exposure assessment, dose–response characterization, and mechanistic understanding. In this narrative review, we integrate current evidence regarding environmental exposure pathways, biodistribution, and molecular mechanisms of MNP-induced toxicity, emphasizing the need for integrated exposome-based approaches, omics technologies, advanced experimental models, and longitudinal studies to improve human health risk assessment and guide regulatory policies.

## 1. Introduction

### 1.1. The Plastic Revolution and Its Environmental Consequences

The advent of plastic production dates to the mid-19th century, when efforts to develop low-cost and versatile substitutes for natural materials such as ivory, horn, and shellac led to the creation of the first semi-synthetic polymers. The creation of Parkesine in 1862 by Alexander Parkes and, later, celluloid by John Wesley Hyatt laid the foundation for modern polymer chemistry [1]. In 1907, Leo Baekeland’s invention of Bakelite marked a milestone as the first fully synthetic plastic, inaugurating a new industrial era characterized by the mass production of lightweight, durable, and moldable materials [2]. Subsequent breakthroughs in polymerization chemistry during and after World War II, notably the development of polyethylene (PE), polypropylene (PP), polystyrene (PS), polyvinyl chloride (PVC), and polyethylene terephthalate (PET), enabled plastics to become ubiquitous across industries ranging from packaging and textiles to electronics, automotive manufacturing, medicine, and aerospace [3,4].

Plastics have also revolutionized modern life due to their versatility, low cost, and exceptional performance-to-weight ratio, catalyzing rapid economic and technological expansion. The global plastic production surpassed 400 million tons in 2023, nearly doubling since the early 2000s, and is projected to triple by 2060 under current consumption trajectories [3,5]. However, only 9% of all plastics ever produced have been recycled, while the remainder have accumulated in landfills, natural ecosystems, or been incinerated, releasing toxic by-products and greenhouse gases [6,7]. Consequently, the environmental persistence of synthetic polymers, often exceeding hundreds of years, has transformed plastic waste into a planetary boundary threat, with plastic debris now detected in virtually all environmental compartments, from oceanic trenches to Arctic snow and atmospheric fallout [8,9].

Beyond solid waste accumulation, the entire life cycle of plastics contributes significantly to the global carbon footprint. From fossil fuel extraction and naphtha cracking to polymerization, product manufacturing, and eventual disposal, plastic production accounts for approximately 4.5% of global greenhouse gas emissions, equivalent to 1.8 billion tonnes of CO_2_ per year [10]. If current trends persist, these emissions could consume up to 15% of the global carbon budget compatible with limiting warming to 1.5 °C by 2050 [11,12]. On the other hand, recent studies highlight that marine microplastics alter phytoplankton productivity, disrupting microbial carbon pumps and impairing biological carbon sequestration. These changes have influenced Earth’s carbon cycling, which is a major natural climate regulator [13,14].

Thus, plastic pollution represents a dual global challenge, it directly threatens ecosystems and biodiversity while indirectly exacerbating climate change and public health risks. Thus, addressing this crisis requires an integrated approach that encompasses material innovation, sustainable production, improved waste management, and policies to reduce plastic dependency across industrial sectors.

### 1.2. From Macro to Nano: Mechanisms of Plastic Fragmentation

Over time, plastic debris undergoes progressive fragmentation into smaller particles through environmental weathering and degradation processes. For consistency throughout this review, we adopt an operational size-based classification in which microplastics (MPs) are defined as plastic particles ranging from 100 nm to 5 mm, whereas nanoplastics (NPs) are defined as particles < 100 nm. We acknowledge that alternative frameworks define nanoplastics as particles < 1 µm; however, the <100 nm threshold is adopted herein to maintain a clear operational distinction between the micro- and nanoscale fractions. Throughout the manuscript, the abbreviation MNPs is used exclusively as a collective term referring to micro- and nanoplastics, whereas MPs and NPs refer specifically to microplastics and nanoplastics, respectively [15]. Unlike many conventional pollutants, these materials exhibit remarkable chemical and physical stability, resisting biodegradation and persisting for decades in environmental matrices [16]. The fragmentation results from combined processes of mechanical abrasion, photodegradation, thermo-oxidation, and hydrolysis, all of which are strongly influenced by environmental factors such as UV radiation, salinity, pH, and temperature fluctuations [17]. In aquatic environments, wave action and sediment friction can contribute to mechanical weathering, whereas in soils, microbial activity and root exudates may facilitate oxidative and enzymatic degradation of polymer chains, contributing to the formation of micro- and nanosized fragments [18,19,20].

The recognition of MPs as a class of pollutants has marked a paradigm shift on environmental science. Although microscopic plastic fibers were first reported in oceanic samples in the 1960s, their ecological significance remained largely overlooked until 2004, when Richard Thompson and colleagues formally defined and popularized the term “microplastics” [21]. Since then, micro- and nanoplastics have been identified in virtually all environmental compartments, such as oceans, rivers, sediments, soils, air, and even polar snow and rainwater, demonstrating their global dispersion and capacity for long-range atmospheric transport [22]. Atmospheric studies have shown that airborne MPs can travel hundreds of kilometers from their emission sources [23], while NP particles, due to their extremely low mass and high surface-area-to-volume ratio, can remain suspended for prolonged periods, facilitating a global-scale circulation [24].

MNPs can originate from primary and secondary sources. Primary particles are deliberately manufactured at microscopic scales for use in industrial applications, personal-care products, paints, and synthetic textiles. For instance, plastic microbeads are commonly found in exfoliating cosmetics and NPs designed for drug delivery or biosensing applications. In contrast, secondary MNPs result from the fragmentation of larger plastic debris, derived from consumer products, fishing gear, tire wear, or packaging waste, as they degrade through physical and chemical stressors [25]. Thus, secondary sources are the main contributors to the environmental burden of MNPs, accounting for most of the MPs load detected in aquatic and terrestrial ecosystems [26,27].

Notably, these particles exhibit an extraordinary diversity of morphologies and polymer chemistry, reflecting their com-plex origins and environmental history. Common shapes include fragments, fibers, films, foams, and spheres, each with distinct aerodynamic and hydrodynamic behaviors that influence transport and deposition patterns [28]. Their polymer composition varies widely, with polyethylene (PE), polypropylene (PP), polystyrene (PS), polyvinyl chloride (PVC), and polyethylene terephthalate (PET) being the most prevalent types [29]. Weathering alters the surface charge, roughness, and hydrophobicity of these particles, promoting the adsorption of metals, organic pollutants, and microorganisms. As a result, they form complex eco-coronas that modulate environmental persistence and interactions with biological membranes [30].

Ultimately, the physicochemical diversity of MNPs governs their environmental behavior, mobility, and bioavailability, as well as their toxicological potential in biological systems [31]. Then, understanding how different environmental stressors drive the transition from macroplastics to nanoscale debris remains crucial for assessing long-term ecological risks and the fate of plastics in the biosphere [32,33].

### 1.3. Human Exposure Routes

**Figure 1:**
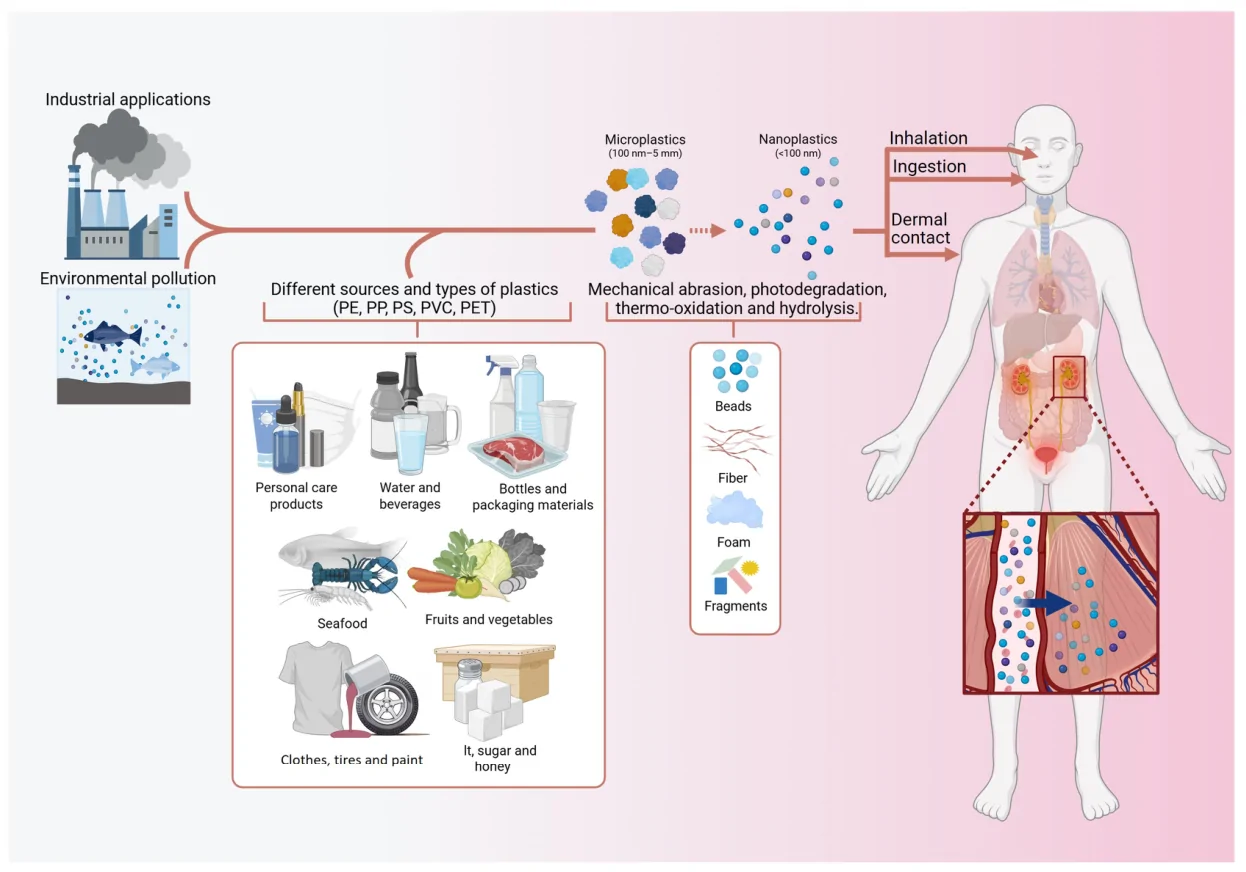
Integrated overview of plastic sources, environmental degradation, human exposure pathways, and renal distribution of micro- and nanoplastics. SOURCES, Plastics are widely used across multiple industrial sectors, including textiles, packaging, automotive, medical, and aerospace applications. Their chemical stability and resistance to biodegradation contribute to contamination of diverse environmental compartments, including oceans, rivers, soils, and air. The most prevalent polymers include polyethylene (PE), polypropylene (PP), polystyrene (PS), polyvinyl chloride (PVC), and polyethylene terephthalate (PET). DEGRADATION, Environmental weathering through mechanical abrasion, photodegradation, thermo-oxidation, and hydrolysis progressively fragments larger plastic materials into microplastics (MPs; 100 nm–5 mm) and nanoplastics (NPs; <100 nm), which may occur as fragments, fibers, foams, or beads. HUMAN EXPOSURE, Humans are exposed to micro- and nanoplastics (MNPs) primarily through ingestion of contaminated food and water, inhalation of airborne particles originating from textiles, tire wear, paints, and household dust, and, to a lesser extent, dermal contact with selected consumer and personal-care products. Following exposure, smaller particles may cross biological barriers and enter the systemic circulation, allowing distribution to internal organs. Given the kidney’s central role in blood filtration and excretion, circulating MNPs can reach the renal compartment, and the presence of plastic particles has been demonstrated in human kidney tissue and urine, supporting the kidney as a relevant target organ for MNP exposure and retention. Created in BioRender. Amaral, M. (2026) https://BioRender.com/jrutdiy.

All types of MNPs are now recognized as ubiquitous contaminants capable of long-range transport by wind and water currents. Humans are exposed to these particles through ingestion, inhalation, and dermal contact, while the environment acts as a reservoir sustaining continuous cycles of contamination [34]. An overview of these exposure routes, their biological interfaces, and their accumulation in organs, such as the kidneys, is illustrated in Figure 1.

Among all exposure routes, ingestion is considered the dominant pathway through which MNPs enter the human body [34,35]. These particles have been detected in drinking water (both bottled and tap), sea salt, honey, beer, seafood, fruits, and vegetables, confirming their pervasiveness in the food chain. For example, one study reported an average of approximately 325 MPs per liter of bottled water (as small as ~6.5 µm), with PP as the most abundant polymer [36]. Studies using microRaman/FT-IR have found much higher particle counts (2000–6000 particles) particularly when smaller size fractions are analyzed [37,38]. Dietary exposure estimates suggest that adults may ingest around 583 ng of plastic particles per day, while children may ingest 184 ng per day, with values varying according to diet and geographic region [39]. Once ingested, NPs (<100 nm) can cross the gastrointestinal barrier through mechanisms including endocytosis or paracellular diffusion into the systemic circulation [40,41]. Furthermore, MNPs can alter gut microbiota composition and induce intestinal inflammation, which may further facilitate their absorption and systemic dissemination [40,42].

Conversely, inhalation is a major but often overlooked route of exposure. MNPs are abundant in both indoor and out-door air, mainly originating from textile fibers, plastic abrasion, and resuspension of household dust [43,44]. Alarmingly, humans may inhale up to 68,000 MPs per day, predominantly in indoor environments where particle density is up to five times higher than outdoors [45]. Once inhaled, the particles can deposit in the upper and lower respiratory tract depending on their aerodynamic diameter, with NPs capable of reaching alveolar regions [46,47]. Moreover, growing evidence suggests that NPs can cross the alveolar epithelium, disseminate via the bloodstream, and accumulate in distant organs [44,47]. Beyond that, occupational environments, such as textile manufacturing and plastic recycling facilities, present higher air-borne concentrations, which are associated with chronic respiratory inflammation and oxidative stress [48].

Although dermal contact is considered a minor route compared to ingestion and inhalation, it remains relevant due to the widespread use of plastics in cosmetics, personal-care products, and medical devices [49]. Primary MPs are intentionally added to formulations such as exfoliating scrubs, toothpaste, and sunscreens, creating repeated skin exposure [50]. While intact skin provides a strong barrier against MPs, NPs (<100 nm) can penetrate through hair follicles, sweat glands, or compromised skin integrity [49,51]. Additionally, cosmetic emulsifiers and surfactants can enhance NP penetration, and once within the epidermis, these particles may interact with immune cells such as Langerhans cells, inducing localized inflammation [50,52].

Collectively, these findings underscore that human exposure to MNPs is continuous, multifactorial, and virtually unavoidable. Ingestion, inhalation, and, to a lesser extent, dermal contact are complementary exposure routes that allow these particles to enter the body, interact with biological barriers, and reach internal tissues. Consequently, the small size and physicochemical diversity, persistence, and bio-adhesive properties of MNPs facilitate their systemic translocation and accumulation in multiple organs. Importantly, the interplay between exposure route, particle characteristics, and host physiology determines the extent and nature of the ensuing biological responses. Understanding these factors is therefore essential to clarify the toxicokinetics of MNPs and their implications for human health.

### 1.4. Mechanistic Insights into MNP Toxicity

Exposure to MNPs has been associated with a wide spectrum of adverse biological effects, including physical damage to cellular and tissue structures, chemical toxicity from leached additives and adsorbed environmental pollutants, and immune activation triggered by oxidative stress and cytokine release [53,54]. In addition, MNPs can disrupt critical biological processes, such as energy metabolism, membrane integrity, protein folding, and gene-expression regulation [55,56]. Collectively, these effects raise serious concerns about the contribution of MNP exposure to chronic inflammation, metabolic dysfunction, and organ injury.

Crucially, the particle–biological interface is dynamic: upon contact with biological fluids, MNPs rapidly acquire an adaptive “eco-/protein corona” composed of environmental macromolecules and host proteins that alter surface chemistry, colloidal stability, and biological identity, thereby modulating cellular recognition, uptake kinetics, and downstream signaling [57,58]. However, the composition and stability of this corona vary according to the local milieu (e.g., plasma or interstitial fluid), particle surface properties, and exposure history, leading to context-dependent toxicological effects [57,59].

In this connection, particle size, shape, surface charge, and polymer type strongly influence translocation and internalization routes [60]. While larger MPs tend to remain extracellular and have been associated with mechanical irritation and barrier dysfunction, NPs can cross epithelial and endothelial barriers via paracellular leakage, receptor-mediated endocytosis, macropinocytosis, and, in some experimental models, transcytosis [61,62]. These uptake mechanisms facilitate systemic distribution, including passage across the blood–brain and placental barriers, and allow interaction with intracellular organelles [60,62].

As an example, once internalized, NPs perturb organelle function through multiple, often inter-related, mechanisms. Of note, a consistent finding among cell lines and animal models is the mitochondrial dysfunction, which is manifested as membrane depolarization, altered dynamics (fission/fusion imbalance), impaired ATP production, and excessive reactive oxygen species (ROS) generation [63,64]. These changes both trigger and amplify oxidative stress signaling cascades [65], whereas the mitochondrial damage contributes to inflammatory activation and can sensitize cells to apoptosis or necroptosis [66].

Concomitantly, NPs often disrupt endo-lysosomal homeostasis by impairing lysosomal acidification, membrane destabilization, or overloading degradative capacity, these alterations can block autophagic flux, promote accumulation of dam-aged proteins/organelles, and activate inflammasome pathways, thereby sustaining sterile inflammation [67,68]. As a result, the lysosomal/autophagy defects are mechanistically linked to genotoxic stress (direct DNA lesions and impaired DNA repair), epigenetic alterations (DNA methylation and histone modifications), and transcriptional reprogramming that together may increase the risk of malignant transformation under conditions of chronic exposure [69,70,71].

Importantly, the multifactorial nature of MNP toxicity and the heterogeneity of reported findings impair the consistency of cross-study comparisons and risk assessment: physicochemical diversity (polymer type, additives, weathering state), formation of different coronas, variability of experimental media, dose metrics (particle number vs. mass), and detection limits for NPs quantification [72,73]. Moreover, human tissue analyses increasingly confirm the presence of MNPs in organs, reinforcing their relevance and under-scoring the urgency of improved analytical methods and standardized toxicological frameworks [74,75]. This is particularly relevant for the kidney, as a major organ of filtration, excretion, and metabolic regulation, which has recently emerged as a key target of MNP bioaccumulation and toxicity. Therefore, this review summarizes current evidence on renal accumulation and toxicity of MNPs, integrating molecular, cellular, and systemic perspectives, and highlighting the implications for kidney disease and potential carcinogenesis.

## 2. Literature Search Strategy

This article was conducted as a narrative review aimed at critically integrating current evidence on micro- and nanoplastics (MNPs) within the human exposome, with particular emphasis on environmental and human exposure pathways, systemic biodistribution, renal accumulation, nephrotoxicity, and potential implications for kidney disease and carcinogenesis.

Relevant literature was identified through searches of the PubMed/MEDLINE database. No initial publication-year restriction was applied to allow the inclusion of seminal studies relevant to the historical and conceptual development of the field, and the literature search was updated through May 2026. Search terms related to plastic particles, including “microplastics”, “nanoplastics”, “micro- and nanoplastics”, and “MNPs”, were combined with terms related to exposure and biological effects, including “human exposure”, “exposome”, “biodistribution”, “bioaccumulation”, “toxicity”, “oxidative stress”, “mitochondrial dysfunction”, “endoplasmic reticulum stress”, “inflammation”, “autophagy”, “fibrosis”, and “carcinogenesis”. To specifically identify kidney-related evidence, these terms were further combined with “kidney”, “renal”, “nephrotoxicity”, “renal accumulation”, “kidney injury”, and “renal cell carcinoma”

Studies were selected according to their relevance to the conceptual, mechanistic, and translational scope of the review. Attention was given to original experimental studies using kidney-derived cell lines, animal models, human tissues and biofluids, and advanced experimental models, including kidney organoids. Studies investigating MNP detection and characterization in human biological samples were also considered. Relevant review articles were included to provide contextual information on environmental sources, exposure routes, physicochemical properties, biodistribution, and established or emerging molecular mechanisms of MNP toxicity.

Duplicate records were identified and removed before the final narrative synthesis. Studies were excluded when they were unrelated to MNP exposure, were outside the biological or renal scope of the review, or did not provide information relevant to the mechanistic or translational questions addressed herein. Because the objective of this work was to provide a critical and mechanistically integrated narrative synthesis rather than a systematic assessment of the available evidence, no formal risk-of-bias assessment or meta-analysis was performed.

## 3. Renal Accumulation and Nephrotoxicity Induced by MNPs

### 3.1. Multilevel Mechanisms of MNP Toxicity: From Cellular Stress to Signaling Interactions

#### 3.1.1. Cellular Uptake and Subcellular Stress Responses

Experimental studies using human kidney-derived cell lines have revealed that PS-MPs and PS-NPs are efficiently internalized by immortalized proximal tubular epithelial cells (HK-2) and embryonic kidney cells (HEK293) within 24–72 h, leading to profound morphological alterations and mitochondrial dysfunction [76,77]. After cellular internalization, MNP exposure triggers excessive generation of reactive oxygen species (ROSs) and mitochondrial depolarization while suppressing key antioxidant enzymes, including superoxide dismutase 2 (SOD2) and catalase (CAT), thereby compromising cellular antioxidant defenses. Inhibition of the heme oxygenase-1 (HO-1) pathway and depletion of glutathione (GSH) further amplify oxidative injury, resulting in apoptosis and autophagy activation [65,78].

Treatment with the mitochondrial-targeted antioxidant MitoTEMPO markedly alleviated these cellular perturbations, underscoring the pivotal role of mitochondrial oxidative stress in mediating plastic particle-induced nephrotoxicity. Mechanistically, exposure to PS-MPs elicited mitochondrial dysfunction, endoplasmic reticulum (ER) stress, inflammatory activation, and autophagic responses in renal tubular cells, which were recapitulated by in vivo findings demonstrating PS-MP accumulation within renal tissues from exposed mice. Collectively, these results provide evidence that sustained exposure to PS-MPs may compromise mitochondrial homeostasis and cellular quality-control mechanisms, contributing to kidney injury [79].

Alongside oxidative imbalance, exposure to PS-MPs has been shown to induce ER stress, leading to activation of unfolded protein response markers and pro-apoptotic cascades. Both in vitro and in vivo experiments demonstrated that PS-MPs elevate ER stress-related proteins and modulate autophagy through Beclin-1 and LC3 pathways [79,80]. Notably, ER stress intersects with mitochondrial ROS signaling to activate caspase-dependent apoptosis. In juvenile rat models, chronic oral exposure to 1 μm PS-MPs for 28 days decreased body weight gain and kidney index, elevated serum creatinine and blood urea nitrogen, and promoted apoptosis through upregulation of Caspase-3, Caspase-9, and Caspase-12 [81]. Co-treatment with antioxidants such as N-acetyl-L-cysteine (NAC) or the ER stress inhibitor Salubrinal alleviated renal inflammation and apoptosis, further supporting oxidative stress-mediated ER injury as an important nephrotoxic mechanism in this experimental model [81].

#### 3.1.2. Inflammatory Signaling, Intercellular Communication, and Fibrotic Remodeling

Beyond subcellular stress responses, inflammatory signaling represents an important component of experimentally observed renal injury. The pro-inflammatory effects of PS-MP exposure are evident across multiple experimental models. PS-MPs activate NF-κB p65, TNF-α, IL-1β, and IL-6 and promote necroptosis through the RIP1/RIP3/MLKL signaling cascade [82]. In mice and avian species, sustained exposure has been associated with tubular damage, macrophage infiltration, interstitial inflammation, and progression toward fibrotic remodeling [83]. Long-term exposure studies demonstrated increased expression of extracellular matrix (ECM)-related and fibrosis-associated genes, as well as tubular epithelial–mesenchymal transition (EMT), associated with TGF-β1 secretion and Wnt/β-catenin pathway activation [84]. Restoration of Klotho protein expression reversed tubular senescence and attenuated fibroblast activation, suggesting the Klotho/Wnt axis as a potential therapeutic target against PS-MP-associated renal fibrotic remodeling [84].

Recent findings further indicate that PS-MPs can disrupt intercellular communication via extracellular vesicles (EVs). PS-MPs enhanced EV release from tubular cells and altered their cargo composition, including Beclin-1 and CD63, which subsequently induced ROS generation and fibrosis-related protein expression in renal fibroblasts [80]. These vesicles may therefore propagate stress signals beyond directly exposed cells, fostering a pro-fibrotic microenvironment within the renal interstitium [80].

#### 3.1.3. Systemic Modifiers and Interorgan Crosstalk

In addition to direct renal effects, systemic and environmental factors may modify the nephrotoxic response to plastic particle exposure. A relevant example of interorgan crosstalk involves the gut–kidney axis, as oral exposure to PS-MPs compromises intestinal barrier integrity, increases circulating complement C5a, and activates the renal C5a/C5aR pathway, leading to inflammation and glomerular dysfunction [85]. Antibiotic-mediated restoration of the gut barrier or pharmacological inhibition of C5aR effectively mitigated kidney injury, supporting a mechanistic link between intestinal permeability, systemic inflammation, and renal complement activation in this experimental model [85]. Importantly, these findings support an indirect gut–kidney inflammatory mechanism but do not establish direct translocation of PS-MPs from the intestinal lumen to the renal circulation or kidney tissue. Thus, in this experimental model, the renal effects should primarily be interpreted in the context of intestinal barrier disruption and complement-mediated systemic signaling rather than as evidence of direct particle trafficking from the gut to the kidney.

The biological response to plastic particles may also be influenced by concomitant inflammatory and metabolic stressors. Co-exposure of PS-NPs with lipopolysaccharide (LPS) markedly aggravated oxidative stress and apoptosis through the IRE1/XBP1 arm of the ER stress pathway, indicating that a pre-existing inflammatory state or co-exposure to additional stressors can amplify renal damage [86]. Moreover, dietary factors such as a high-fat diet (HFD) exacerbate PS-MP-induced renal injury, driving extracellular matrix remodeling, ROS-associated carcinogenic signaling, and enrichment of profibrotic PF4+ macrophages [87]. Together, these findings indicate that MNP toxicity is context-dependent and may be modified by metabolic and inflammatory conditions.

#### 3.1.4. Renal Accumulation, Toxicokinetics, and Human Translational Evidence

At the tissue level, rodent studies consistently reveal MNP bioaccumulation within renal tissues, with evidence of particle retention, histopathological injury, and elevated renal biomarkers, including blood urea nitrogen (BUN), creatinine, and uric acid, following chronic oral exposure [83,88]. These findings provide experimental evidence that renal exposure is influenced not only by cellular toxicity but also by particle distribution and retention within the kidney.

Importantly, human evidence currently provides information primarily on particle detection and renal distribution rather than nephrotoxicity or disease causation. A landmark microRaman spectroscopy study confirmed the presence of MPs in human kidney tissue and urine, identifying polyethylene and polystyrene fragments ranging from 1 to 29 μm [89]. These observations provide direct evidence that MPs can be detected within the human renal system and urinary compartment; however, they do not establish the route by which these particles reach the renal parenchyma or urine. In particular, particles within this micrometer-size range greatly exceed the dimensions generally compatible with conventional glomerular filtration and therefore should not be assumed to cross the glomerular filtration barrier by passive filtration. Their detection in kidney tissue likewise does not establish renal toxicity or disease causation.

From a toxicokinetic perspective, subsequent reviews have proposed a filtration–reabsorption–translocation framework for renal MNP handling [90,91]. However, this model should be interpreted as strongly size-dependent. The glomerular filtration barrier acts as a nanoscale sieve, with an overall size cutoff of approximately 8–10 nm for conventional nanoparticle filtration, although renal passage is also influenced by hydrodynamic size, surface charge, shape, and particle deformability [92]. Accordingly, micrometer-sized particles, including the 1–29 μm MPs detected in human renal samples [89], exceed this filtration threshold by several orders of magnitude and should not be assumed to access the tubular lumen through conventional passive glomerular filtration. Their presence within renal tissue may instead reflect alternative processes following systemic distribution, potentially including interactions with the renal microvascular endothelium and active transendothelial or endocytic transport pathways. Nevertheless, direct evidence for these mechanisms in environmental MNP exposure remains limited, particularly in humans. Thus, the filtration–reabsorption–translocation framework should be regarded as particle-size-dependent rather than as a universal mechanism governing renal MNP handling, and the pathways responsible for the renal localization, retention, and urinary occurrence of larger MPs remain insufficiently characterized.

**Figure 2:**
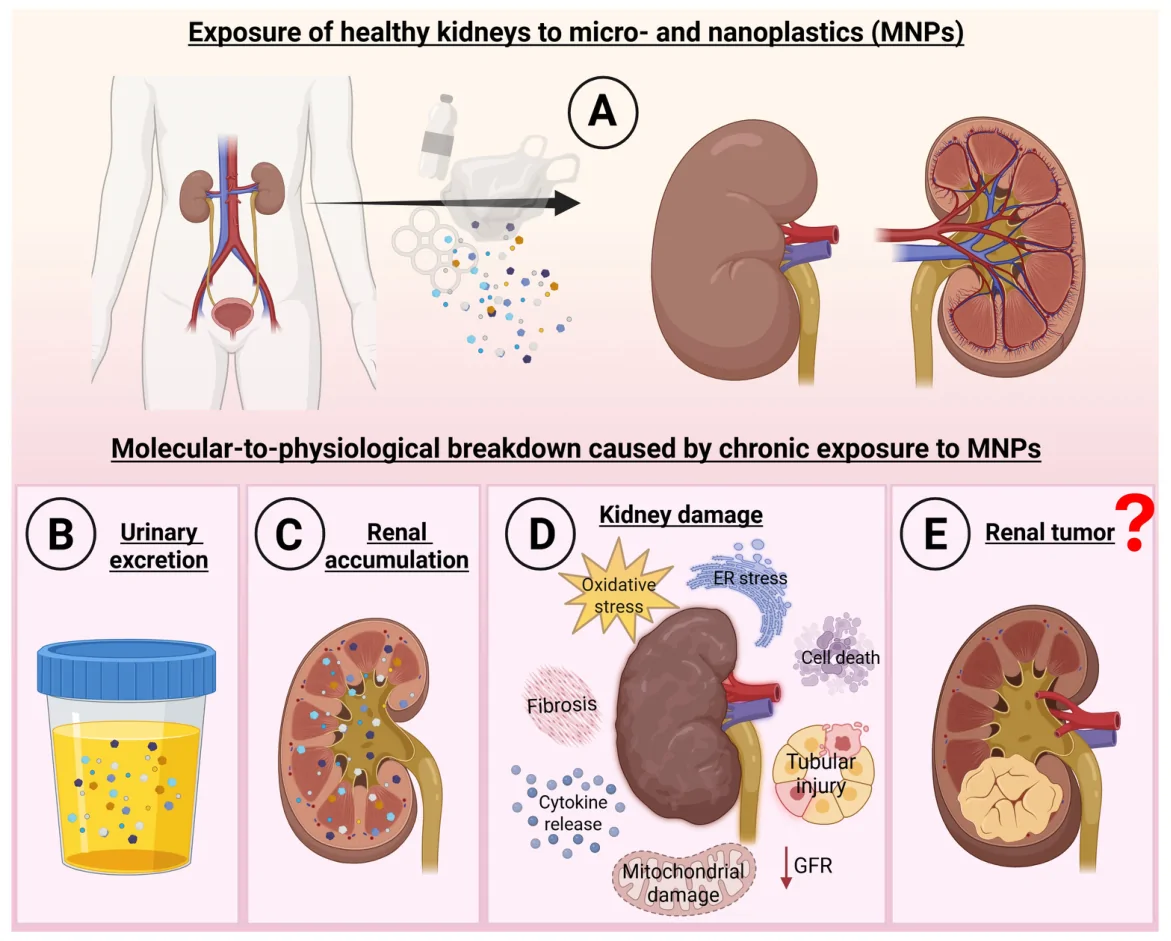
Renal exposure to micro- and nanoplastics: evidence-based mechanisms of kidney injury and emerging implications for carcinogenesis. (**A**) Under physiological conditions, the kidney maintains fluid and electrolyte homeostasis through blood filtration, solute reabsorption, and metabolite excretion. (**B**) Micro- and nanoplastics (MNPs) can reach the renal system, as demonstrated in experimental models. In humans, MPs have been detected in urine, indicating that at least a fraction of these particles can be eliminated through the urinary tract. Persistent exposure and/or reduced clearance may favor particle retention within renal tissue. (**C**) Experimental studies support renal accumulation or retention of MPs and NPs, whereas human tissue analyses have demonstrated the presence of MPs in the renal compartment. These findings support renal exposure, while the biological consequences of particle retention in humans remain to be established. (**D**) MNP-associated renal injury is supported by experimental evidence demonstrating mitochondrial dysfunction, endoplasmic reticulum stress, oxidative stress, inflammatory signaling, tubular injury, cell death, and fibrotic remodeling. Collectively, these alterations have also been associated with the activation of signaling pathways relevant to pro-oncogenic cellular remodeling. (**E**) The potential relationship between chronic MNP exposure and renal carcinogenesis remains an emerging area of investigation. The question mark (?) indicates an uncertain and not yet established association. Although MNP-associated oxidative and genotoxic stress, chronic inflammation, mitochondrial dysfunction, metabolic reprogramming, and pro-oncogenic signaling provide biological plausibility for a potential link, there is currently insufficient evidence to establish a direct causal relationship between MNP exposure and kidney tumor development. Further mechanistic, epidemiological, and longitudinal studies are required to clarify this potential association. Created in BioRender. Foresto Neto, O. (2026) https://BioRender.com/eo6b9hr.

Together, these findings outline a multilevel framework of renal effects associated with MNP exposure, progressing from cellular uptake and subcellular stress to inflammatory signaling, maladaptive repair and fibrotic remodeling, and systemic crosstalk. Experimental models provide evidence for several of these biological effects, whereas current human evidence primarily demonstrates MP presence in renal tissue and urine. Thus, Figure 2 provides an integrated overview of the mechanisms associated with renal MNP exposure while distinguishing experimentally demonstrated biological responses from emerging translational implications.

### 3.2. Renal Exposure to MNPs and Oncogenic Pathways

Building on the mechanisms described above, chronic renal inflammation, oxidative stress, and fibrotic remodeling are well-established features associated with tumor-promoting microenvironments and the pathogenesis of renal cell carcinoma (RCC) and urothelial malignancies [93,94]. Given that experimental studies have reported MNP-associated DNA damage, alterations in DNA-repair mechanisms, and changes in epigenetic marks, these findings provide biological plausibility for investigating a potential relationship between MNP exposure and renal carcinogenesis [95,96]. However, such mechanistic overlap should not be interpreted as evidence that MNP exposure causes renal cancer in humans.

In experimental cellular models, NPs have been reported to induce DNA double-strand breaks and micronucleus formation and to modulate cancer-related transcription factors and signaling pathways, including c-MYC, HIF-1α, NF-κB, and MAPK. Moreover, MNP-associated metabolic alterations, including increased reliance on glycolytic metabolism, have been described in experimental systems and overlap with metabolic features frequently observed in malignant cells. Together, these observations identify molecular processes of potential relevance to tumor biology but do not establish malignant transformation or carcinogenesis. Human evidence remains particularly limited and currently does not demonstrate an epidemiological or causal association between MNP exposure and renal cancer. Importantly, co-exposure to MNP-associated or adsorbed environmental contaminants with known or suspected carcinogenic properties, including bisphenol A, polycyclic aromatic hydrocarbons (PAHs), and heavy metals, may further modify toxicological responses; however, the relevance of these combined exposures to human renal carcinogenesis remains to be established [97,98,99].

Collectively, current evidence supports a mechanistic hypothesis rather than a causal relationship between MNP exposure and renal malignancy. The convergence of oxidative and genotoxic stress, chronic inflammation, mitochondrial dysfunction, metabolic reprogramming, and epigenetic alterations observed across experimental models reflects biological processes that also participate in tumor-associated pathways. This overlap provides a rationale for further investigation of MNPs within the context of environmental oncology, particularly in the kidney, where human studies have demonstrated the presence of plastic particles in renal tissue and urine. Nevertheless, detection of MPs in the human renal compartment demonstrates exposure and tissue distribution, not disease causation. Whether chronic exposure, renal retention, or MNP-induced molecular alterations contribute to the initiation or progression of renal malignancies remains unknown and will require dedicated mechanistic studies together with longitudinal and epidemiological evidence. In this context, Table 1 summarizes the current experimental and human evidence regarding MNP exposure and renal toxicity, while highlighting the distinction between demonstrated biological effects and emerging mechanistic associations.

### 3.3. Pathophysiological Integration and Translational Implications

Evidence increasingly supports that MNPs elicit a complex and multifactorial nephrotoxic response driven by their bioaccumulation within renal tissue [90,100]. Once internalized, MNPs initiate a cascade of deleterious processes characterized by mitochondrial dysfunction, oxidative and ER stress, inflammation, and fibrogenesis [80,101]. These cellular perturbations converge to disrupt renal homeostasis and promote maladaptive repair responses that may culminate in chronic injury. Importantly, MNP-induced nephrotoxicity extends beyond isolated cellular effects, reflecting a systems-level disturbance involving multiple signaling networks and organ crosstalk [102].

A growing body of evidence underscores the relevance of cross-organ communication in MNP-mediated renal pathology. The gut–kidney axis appears to be a major conduit, whereby intestinal barrier disruption and microbial dysbiosis amplify renal oxidative stress and inflammation through immune and complement activation [90,100]. Similarly, EVs released by tubular cells exposed to polystyrene MPs have been shown to propagate stress signals, driving ER stress and fibrosis in neighboring fibroblasts [80]. In parallel, maladaptive stress responses such as autophagy, necroptosis, and ferroptosis emerge as critical mediators of tubular cell death and interstitial fibrosis, linking MNP exposure to progressive loss of renal function [101,102].

The detection of MPs in human kidneys represents a landmark finding, confirming that these particles can not only reach but also persist within the renal parenchyma [90]. Such bioaccumulation raises significant concerns regarding their contribution to the global burden of CKD and renal fibrosis, particularly given the ubiquity of environmental exposure [100].

Nonetheless, the field still faces major knowledge gaps. The kinetics of MNP clearance, their size- and charge-dependent renal tropism, and their synergistic interactions with metabolic, inflammatory, or infectious comorbidities remain poorly understood. Moreover, inconsistencies in exposure models, particle characterization, and dosage regimens hinder the comparability of studies and obscure translational interpretation. Addressing these challenges requires standardized experimental protocols that reflect realistic environmental exposure levels and leverage advanced mechanistic tools to dissect the molecular underpinnings of plastic-induced nephrotoxicity. Bridging these mechanistic insights with translational approaches is the next crucial step toward defining the real-world health burden of renal MNP exposure.

## 4. Future Directions: From Mechanistic Insight to Clinical Translation

Advancing from descriptive toxicology to mechanistic and translational understanding of MNP-induced human diseases requires an integrated, interdisciplinary framework. A critical priority is the standardized quantification of MNPs in human tissues and biofluids through high-resolution analytical platforms, such as micro-Raman and Fourier-transform infrared spectroscopy, complemented by mass-based and isotopic imaging to map bioaccumulation dynamics in vivo. In this context, intracellular accumulation during repeated or chronic exposure has been proposed as an additional toxicokinetic consideration for NPs; however, whether such accumulation results in tissue concentrations comparable to those associated with adverse effects in experimental systems remains uncertain and requires quantitative investigation [103].

In parallel, longitudinal population-based studies are essential to establish causal relationships between cumulative exposure, internal MNP load, and disease progression, particularly among environmentally vulnerable populations. At the molecular level, comprehensive multi-omics profiling, including transcriptomics, epigenomics, metabolomics, and proteomics, should be applied to define exposure-specific molecular signatures and identify early biomarkers of MNP-induced toxicity. Such system-level approaches can uncover novel mechanistic pathways underlying renal and systemic injury. Furthermore, advanced experimental platforms such as human tissue organoids, microfluidic organ-on-chip systems, and robust in vivo models, integrated with artificial intelligence-driven toxicogenomic pipelines, offer unprecedented opportunities to replicate human micro-physiology and validate mechanistic hypotheses under controlled yet physiologically relevant conditions.

Ultimately, the integration of experimental, epidemiological, and computational evidence will be critical to develop a coherent risk assessment framework for MNP exposure. This translational bridge will not only refine our understanding of the molecular pathology of MNP-induced complex disease but also guide evidence-based regulatory policies and preventive strategies aimed at mitigating the long-term consequences of plastic pollution in human populations. Only by integrating these experimental, epidemiological, and computational efforts can we transform the current descriptive toxicology of MNPs into actionable biomedical insight.

## 5. Conclusions and Perspectives

Micro- and nanoplastics (MNPs) have emerged as biologically active and persistent components of the human exposome, with the capacity to cross physiological barriers, reach distant organs, and disrupt cellular homeostasis through oxidative, inflammatory, and genotoxic mechanisms. Within experimental renal models, MNP exposure has been associated with a complex network of molecular disturbances, including mitochondrial and lysosomal dysfunction, redox imbalance, inflammatory signaling, and fibrotic remodeling. These processes overlap with mechanisms implicated in chronic kidney injury and tumor-associated biology; however, whether chronic MNP exposure contributes to the development or progression of chronic kidney disease (CKD), renal fibrosis, or renal carcinogenesis in humans remains to be established.

Several unresolved questions currently limit the translation of experimental findings into an understanding of human renal risk. A central challenge is determining how particle-specific properties, including size, polymer composition, surface characteristics, and environmental transformation, influence renal access, cellular uptake, retention, and clearance.

An additional translational limitation is the predominance of pristine, spherical polystyrene (PS) particles in experimental studies. Such standardized particles facilitate controlled mechanistic investigation but do not fully reproduce the physicochemical heterogeneity of environmental MNPs, which may include irregular fragments, fibers, and films composed of polymers such as polyethylene (PE), polypropylene (PP), and polyethylene terephthalate (PET), often subjected to environmental weathering. Differences in particle morphology, polymer composition, surface chemistry, aging, and associated additives or contaminants may influence cellular interactions, uptake, biodistribution, retention, and toxicity. Environmental weathering may also promote the formation of complex eco-coronas through the adsorption of biomolecules, microorganisms, metals, and organic contaminants, further modifying particle surface properties and biological interactions. Therefore, findings obtained with pristine PS particles should not be directly generalized to the complex mixtures encountered under real-world exposure conditions, highlighting the need for studies using environmentally representative particles, weathered materials, eco-corona-bearing particles, and polymer mixtures.

### Dose–Response Disconnect and Gaps in Human Risk Assessment

A major limitation in translating experimental MNP toxicology to human health is the substantial disconnect between the exposure conditions used in mechanistic studies and those expected under real-world scenarios. Experimental studies frequently rely on relatively short exposure periods, comparatively high particle concentrations, and well-defined model particles, whereas human exposure is likely to be chronic, heterogeneous, and composed of complex mixtures. Whether the molecular responses observed under experimental conditions persist at environmentally relevant exposure levels, accumulate over time, or are reversible after exposure cessation remains uncertain. Importantly, the available evidence does not currently establish whether internal particle burdens in humans reach concentrations associated with adverse effects in experimental systems. Resolving these questions will require experimental designs that more closely reproduce realistic exposure scenarios and distinguish transient cellular stress responses from persistent maladaptive alterations.

Equally important is the gap between evidence of human exposure and evidence of human disease. The detection of plastic particles in renal tissue or urine demonstrates that the kidney can encounter these contaminants but does not establish that particle burden predicts renal dysfunction, CKD progression, fibrosis, or carcinogenesis. Population-level exposure–response relationships for renal outcomes remain largely undefined, and the available human evidence is insufficient to determine whether increasing external exposure or internal particle burden is associated with progressive molecular, functional, histopathological, or clinical alterations. Thus, the biological effects identified in experimental systems should currently be interpreted primarily as evidence of potential hazard rather than established human renal risk. Future studies should move toward quantitatively testable relationships between particle exposure and biological outcomes, integrating validated particle characterization with renal functional parameters, molecular biomarkers, histopathological alterations, and longitudinal clinical data.

A major barrier to such risk assessment is the absence of a quantitative framework linking external MNP exposure, internal dose, renal particle burden, biological response, and adverse outcome. The substantial heterogeneity in particle size, polymer composition, surface characteristics, exposure metrics, experimental concentrations, exposure duration, biological models, and analytical approaches limits comparisons across studies and currently prevents environmentally relevant exposure levels from being reliably translated into renal tissue concentrations or thresholds for adverse effects. Consequently, baseline internal exposure levels associated with normal environmental exposure and thresholds associated with adverse renal effects in human populations remain undefined. Establishing this external exposure–internal dose–renal burden–response continuum should therefore represent a major priority for future toxicological and epidemiological research.

Another important but insufficiently explored dimension is whether established determinants of renal vulnerability modify biological responses to MNP exposure. Diabetes, hypertension, chronic inflammatory conditions, and exposure to nephrotoxic drugs are major contributors to kidney injury, yet their potential interactions with MNP exposure remain poorly characterized. This represents an important knowledge gap, particularly because pre-existing metabolic, vascular, or inflammatory stress may influence particle handling or the renal response to additional environmental insults. Future studies should therefore incorporate clinically relevant comorbidities and co-exposures into experimental and epidemiological designs to determine whether established renal risk factors modify susceptibility to MNP-associated effects, rather than assuming that responses observed in otherwise healthy models are uniformly applicable across populations.

Addressing these limitations will require greater methodological harmonization across environmental, experimental, and human studies. Improved analytical methodologies for the detection and quantitative characterization of MNPs in human biofluids and tissues, including advances in microspectroscopic and mass-based imaging technologies, will be essential for defining internal particle burden and mapping biodistribution kinetics. These approaches should be combined with standardized particle characterization and exposure metrics, environmentally relevant chronic-exposure models, and assessment of the persistence or reversibility of biological responses after exposure cessation. Integrating these measurements with molecular, computational, histopathological, renal functional, and epidemiological data may ultimately allow the field to progress from evidence of exposure and experimental hazard toward quantitative assessment of human renal risk.

Ultimately, the central question is not simply whether MNPs can reach the kidney or perturb renal cells under experimental conditions, but under which exposure conditions, particle characteristics, and host susceptibility contexts these interactions become persistent and clinically meaningful. Resolving this question will require closer integration of environmental exposure science, analytical chemistry, experimental toxicology, nephrology, and population-based research. Such evidence will be essential to determine the long-term significance of MNP exposure for human renal health and to support scientifically grounded risk-assessment strategies and preventive public health policies.

## Figures and Tables

**Table 1:** Summary of experimental and clinical evidence on renal toxicity induced by MNPs.

Model/Exposure	Particle Characteristics	Main Findings	Key Biomarkers/Pathways	Evidence Context/Considerations	Reference
HEK293 kidney cells exposed to 1 μm polystyrene MPs (up to 100 μg/mL, 24–72 h)	Polystyrene MPs, 1 μm	High internalization (>70% cells) and morphological changes; reduced proliferation but preserved viability; increased ROS generation; impaired antioxidant defense; altered cellular metabolism.	↑ ROS; ↓ SOD2; ↓ CAT; ↓ GAPDH.	In vitro study using a single polymer type, findings provide mechanistic evidence under controlled experimental conditions.	[76]
HEK293 kidney cells exposed to polystyrene MPs 0.003–0.300 μg/mL.	Polystyrene MPs, round, 3.54 ± 0.39 μm	Cellular internalization; oxidative stress via HO-1 inhibition; mitochondrial depolarization; concurrent apoptosis + autophagy; dose-dependent inflammatory modulation; impaired kidney barrier integrity; potential risk of AKI.	↓ HO-1, ↓ mitochondrial membrane potential, ↑ autophagy, ↑ apoptosis, cytokine changes (33–35), ↓ NLRP3, ↓ ZO-2, ↓ α1-antitrypsin.	In vitro study using a single polymer type, provides mechanistic evidence at the cellular level.	[77]
HK-2 human proximal tubular cells; C57BL/6 male mice exposed to PS-MPs (in vitro and oral gavage)	Polystyrene microplastics (PS-MPs), 2 μm; hydrodynamic size, PDI, and zeta potential characterized by DLS; morphology and particle size confirmed by TEM.	PS-MP uptake induces mitochondrial ROS, ER stress, inflammation, and autophagy; increases Bad protein; alters MAPK and AKT/mTOR pathways; mitochondrial ROS scavenger (MitoTEMPO) reverses effects; in mice, PS-MPs accumulate in kidney and cause histopathological lesions, ER stress, inflammation, and autophagy activation.	↑ Mitochondrial ROS; ↑ Bad; ↑ ER-stress markers; ↑ LC3; ↑ Beclin-1; ↑ inflammatory markers; MAPK alterations; AKT/mTOR signaling changes.	Combined in vitro and in vivo experimental study using PS model particles, provides mechanistic and renal histopathological evidence.	[79]
Juvenile rats orally exposed to PSMPs (1000 nm; 2 mg/kg/day for 28 days)	Polystyrene microplastics (PSMPs), 1 μm (1000 nm)	Reduced body and kidney growth indices; kidney histological lesions; increased oxidative stress, inflammation, and ER stress; intestinal injury; apoptosis activation; NAC and Salubrinal attenuate oxidative stress, ER stress, inflammation, and apoptosis.	↑ BUN, ↑ creatinine; ↑ IL-1β, ↑ IL-6, ↑ TNF-α; ↑ ER stress markers; ↑ apoptosis markers (Bcl-2, Bax, Caspase-12/9/3); ↑ TUNEL-positive cells; oxidative-stress-mediated ER stress.	In vivo juvenile rat model with controlled oral exposure to PS-MPs for 28 days; provides evidence of renal injury and associated oxidative and ER stress responses.	[81]
Chickens orally exposed to PS-MPs (1, 10, 100 mg/L for 6 weeks)	Polystyrene microplastics (PS-MPs), 5 μm.	Mitochondrial structural damage and altered dynamics (fusion/fission imbalance); oxidative stress due to disrupted antioxidant enzymes; renal tissue damage and inflammation; activation of necroptosis signaling (RIP1/RIP3/MLKL); dose-dependent renal injury.	Mitochondrial dynamics markers (MFN1/2, OPA1, Drp1); antioxidant markers (SOD, CAT, MDA, GSH, T-AOC); ↑ NF-κB p65; ↑ TNF-α, ↑ iNOS, ↑ IL-1β, ↑ IL-6; necroptosis pathway: ↑ RIP1/RIP3/MLKL.	Avian in vivo model evaluating concentration-dependent renal effects under controlled exposure conditions.	[82]
Mice exposed chronically to MPs of different sizes (80 nm, 0.5 µm, 5 µm)	Microplastics (MPs), 80 nm, 0.5 µm, 5 µm	Size-dependent kidney injury; induction of inflammation, oxidative stress, and apoptosis; progression toward kidney fibrosis; transcriptomic alterations linked to immune response (80 nm) and circadian rhythm disruption (0.5–5 µm).	↑ Inflammatory response; ↑ oxidative stress markers; ↑ apoptosis; fibrosis-related pathways; gene expression changes in immune pathways (80 nm) and circadian rhythm genes (0.5 and 5 µm).	In vivo model comparing multiple particle sizes, provides size-dependent mechanistic and transcriptomic evidence of renal injury and fibrosis.	[83]
Mice exposed for 28 days to PS, PS-SO_3_H, and PS-NH_2_ MPs (human-equivalent dose)	Polystyrene microplastics (PS-MPs), 5 μm; unmodified PS, amino-modified PS-NH_2_, and sulfonic-modified PS-SO_3_H.	Increased serum kidney injury markers; proteinuria and microalbuminuria; persistent renal inflammation and fibrosis driven by tubular epithelial cell senescence; activation of fibroblasts via TGF-β1; epithelial–mesenchymal transition promoted by altered Klotho/Wnt/β-catenin signaling.	↑ UREA, ↑ BUN, ↑ CREA, ↑ uric acid; ↑ urine protein & microalbumin; ↑ inflammation; ↑ TGF-β1; ↑ fibrosis markers; ↑ cell senescence; Klotho/Wnt/β-catenin pathway dysregulation.	In vivo study comparing PS particles with defined surface modifications, enabling assessment of surface chemistry-dependent effects.	[84]
Human tubular epithelial cells and fibroblasts exposed to polystyrene MPs; in vivo confirmation (mouse urine EV markers)	Polystyrene microplastics (PS-MPs; C37278, Invitrogen); particle size not explicitly reported in the study.	PS-MPs increase EV production in tubular cells; induce ER stress (without classical inflammation) in tubular cells; conditioned medium from PS-MP-treated tubular cells triggers ROS generation, ER stress, and fibrosis-related proteins in fibroblasts; EV-mediated communication drives fibrotic signaling.	↑ EV markers (incl. CD63), ↑ Beclin-1; ↑ ROS; ↑ ER-stress proteins; ↑ fibrosis markers in fibroblasts; EV-mediated signaling.	Mechanistic in vitro study of EV-mediated tubular cell–fibroblast communication, complemented by in vivo assessment of urinary EV markers.	[80]
Mice orally exposed to polystyrene microplastics (PS-MPs)	Polystyrene microplastics (PS-MPs), 2 μm, plain microspheres.	PS-MPs impair gut barrier integrity, elevate urinary C5a, and increase renal C5aR expression, resulting in kidney injury; kidney damage mitigated by antibiotic-induced restoration of gut barrier; C5aR inhibition confirms pathway’s causal role; highlights gut–kidney axis involvement.	↑ C5a (urine), ↑ renal C5aR; gut barrier disruption; C5a/C5aR inflammatory pathway activation; improvement with antibiotics or C5aR inhibitor PMX53.	In vivo oral exposure model investigating gut–kidney axis involvement, with pharmacological targeting of C5aR supporting the mechanistic role of C5a/C5aR signaling.	[85]
Mice and HEK293 cells exposed to polystyrene nanoplastics (PS-NPs), lipopolysaccharide (LPS), or combined PS-NPs + LPS.	Polystyrene nanoplastics (PS-NPs).	PS-NPs aggravate LPS-induced renal apoptosis through enhanced oxidative stress and ER stress; co-exposure triggers stronger activation of the IRE1/XBP1 pathway and promotes apoptosis; antioxidants or ER-stress inhibitors reduce these effects, indicating oxidative stress as the initiating event.	↑ Oxidative stress; activation of IRE1/XBP1 ER-stress pathway; ↑ Caspase-3 and ↑ Caspase-12 (apoptosis markers); inhibition by N-acetyl-L-cysteine and 4-phenylbutyric acid confirms mechanism.	Combined in vitro and in vivo co-exposure model, enabling mechanistic assessment of PS-NP interactions with LPS-induced inflammatory and cellular stress responses.	[86]
Mice exposed to polystyrene microplastics (PS-MPs), high-fat diet (HFD), or combined PS-MPs + HFD for 35 days; kidney analyzed by single-cell RNA sequencing.	Polystyrene microplastics (PS-MPs), 1 μm, spherical.	Combined PS-MPs + HFD worsens kidney injury, induces a profibrotic and pro-tumorigenic microenvironment, disrupts renal epithelial development, and promotes ROS-driven carcinogenic signatures. Treatment activates PI3K-Akt, MAPK, and IL-17 pathways in endothelial cells, increases CD8^+^ effector and proliferating T cells, and remodeling of mononuclear phagocytes, including PF4^+^ M2-like macrophages associated with fibrosis and carcinogenesis.	Extracellular matrix remodeling; ↑ ROS; activation of PI3K-Akt, MAPK, IL-17 signaling; ↑ PF4^+^ macrophages; ↑ oxidative phosphorylation and chemical carcinogenesis pathways; immune shifts (↑ CD8^+^ effector T cells, ↑ proliferating T cells).	In vivo combined-exposure model integrating PS-MPs and HFD, with single-cell transcriptomic analysis enabling cell-type-specific assessment of renal responses.	[87]
Human kidney tissues (healthy regions from nephrectomies) and urine samples from healthy donors; micro-Raman spectroscopy used to detect microplastics.	Polyethylene and polystyrene microplastics; pigments including hematite and Cu-phthalocyanine; size range: 1–29 μm (kidney) and 3–13 μm (urine).	Study provides the first direct evidence of microplastic deposition in human kidneys and confirms elimination via urine. Development and validation of an open-access spectral comparison software improved detection accuracy. Findings indicate that microplastics can accumulate in renal tissue in humans, raising concerns about chronic exposure and potential long-term renal effects.	Not mechanistic—analytical detection study. Relevant identifiers include Polymer types: polyethylene, polystyrene. Pigments: hematite, Cu-phthalocyanine. Physical characteristics: particle presence, size distribution, and spectral profiles.	Human analytical detection study providing direct evidence of microplastic occurrence in kidney tissue and urine; the study design assesses particle presence and characteristics rather than renal toxicity or disease outcomes.	[89]

Notes: 1. Experimental doses are reported as described in the original studies. Direct comparison with estimated human exposure is currently limited by differences in dose metrics and by the absence of validated relationships between external exposure, internal dose, renal particle burden, and adverse-effect thresholds. 2. Exposure concentrations are presented using standardized mass-concentration units whenever possible. Particle-number concentrations were retained only when reported by the original studies and were not retrospectively calculated when unavailable, because such conversions depend on particle density, size distribution, morphology, and aggregation state. [↑ indicates increased expression, and ↓ indicates decreased expression of the analyzed targets].

## Data Availability

No new data were created or analyzed in this study. Data sharing is not applicable to this article.

## Abbreviations

The following abbreviations are used in this manuscript:

AKI	Acute Kidney Injury
ARE	Antioxidant Response Element
BUN	Blood Urea Nitrogen
CAT	Catalase
CKD	Chronic Kidney Disease
CREA	Creatinine
ER	Endoplasmic Reticulum
EVs	Extracellular Vesicles
GSH	Glutathione
HFD	High-Fat Diet
HO-1	Heme Oxygenase 1
IRE1	Inositol-Requiring Enzyme 1
MAPK	Mitogen-Activated Protein Kinase
MDA	Malondialdehyde
MLKL	Mixed Lineage Kinase Domain-Like Protein
MNPs	Micro(nano)plastics
MPs	Microplastics
mTOR	Mechanistic Target of Rapamycin
NAC	N-Acetylcysteine
NLRP3	NOD-, LRR- and Pyrin Domain-Containing Protein 3
PET	Polyethylene Terephthalate
PE	Polyethylene
PI3K	Phosphoinositide 3-Kinase
PMX53	C5a Receptor Antagonist
PP	Polypropylene
PS-MPs	Polystyrene Microplastics
PS-NPs	Polystyrene Nanoplastics
PVC	Polyvinyl Chloride
RIP1	Receptor-Interacting Protein Kinase 1
RIP3	Receptor-Interacting Protein Kinase 3
ROS	Reactive Oxygen Species
SOD	Superoxide Dismutase
T-AOC	Total Antioxidant Capacity
TGF-β1	Transforming Growth Factor Beta 1
UREA	Urea
XBP1	X-Box Binding Protein 1
